# 

**Published:** 1842-05

**Authors:** 


### CONTENTS

OF NO. V.

---

### ORIGINAL COMMUNICATIONS.

ESSAYS AND CASES.

Art. I.—Observations on the epidemic Yellow Fever of Natchez, and of the South-west. By John W. Monette, M. D., of Washington, Mississippi. - - 321

Art. II.—Cesarean Operation terminating fatally. By John Travis, M. D., of Washington, Mississippi. 352

BIBLIOGRAPHICAL NOTICES.

Art. III.—Report on the Establishment of an Asylum for the Insane, made to the Legislature of Indiana, during the Session of 1841–2. - - - - - 355

Art. IV.—Proceedings of the Medical Convention of Ohio, held at Columbus, on the 5th, 6th, and 7th of May, 1841: with several papers read before that body. Columbus, Wright and Legg, 1841, pp. 84. 357

SELECTIONS FROM AMERICAN AND FOREIGN JOURNALS.

Anchylosis of the Lower Maxilla with the Temporal Bones. 367

Extraction of Foreign Bodies from the Ear by Syringing with Cold Water. - - - - - - 368

New Variety of Hernia—Hernia destitute of Peritoneal Sac. 369

Cure of Ozœna. - - - - - - 369

Regeneration and Union of Nerves. - - - 370
Experimental Researches on the Function of the Skin in Man and Animals. - - - - - 371
On Fibre. - - - - - - 372
Fatal Hæmorrhage from the Extraction of a Tooth. - 377
The Structure of the Human Placenta. - - - 379
Supposed Polypus Nasi. - - - - 381
Quinine in Asthma. - - - - - 382
The Sounds of the Heart. - - - - 383
Nitrate of Silver and Ipecacuanha in Diarrhœa and Dysentery. 383
Rectifications in the Practice of Auscultation and Percussion. 383
Ioduret of Sulphur in Porrigo. - - - - 388
Pharmacy in France. - - - - - 389
Death from Erysipelas. - - - - - 391

ORIGINAL INTELLIGENCE.

The past Winter and present Spring. - - - 393
Progress of Total Abstinence. - - - - 394
The Sanicula Marilandica a Cure for the Bite of a Snake 394
Operation for Club Foot. - - - - 395
French Measles. - - - - - 397
Dry Grangrene. - - - - - 397
Flowers of Sulphur and Cream of Tartar and Magnesia in Dysentery. - - - - - - 398
Cold Dash in the Asphyxia of Infants. - - - 398
Leaden Canula in the Treatment of Fistula in Ano. - 399
Liston's Elements of Surgery—new edition. - - 399
New Works on Botany. - - - - 400
Correction. - - - - - - 400

